# Proteomic Analysis Based on TMT Regarding the Therapeutic Action of *Rhizoma Drynariae* on Rats in an Osteoporosis Model

**DOI:** 10.2174/0113862073261905231110061401

**Published:** 2023-11-27

**Authors:** Hui Su, Binghan Yan, Ruochong Wang, Zhichao Li, Zhanwang Xu, Haipeng Xue, Guoqing Tan

**Affiliations:** 1 Shandong University of Traditional Chinese Medicine, Jinan, Shandong, China;; 2 Affiliated Hospital of Shandong University of Traditional Chinese Medicine, Jinan, Shandong, China;; 3 Beijing University of Traditional Chinese Medicine, Beijing, China

**Keywords:** *Rhizoma drynariae*, therapeutic action, TMT, rats, osteoporosis, proteomic analysis

## Abstract

**Background:**

Primary osteoporosis has increasingly become one of the risk factors affecting human health, and the clinical effect and action mechanism of traditional Chinese medicine in the treatment of primary osteoporosis have been widely studied. Previous studies have confirmed that in traditional Chinese medicine (TCM), *Drynaria rhizome* has a role in improving bone density. In this study, a tandem mass tag (TMT)-based proteomic analysis was conducted to derive potential targets for *Drynaria rhizome* treatment in postmenopausal osteoporosis.

**Methods:**

The model group (OVX) and experimental group (OVXDF) for menopausal osteoporosis were established using the universally acknowledged ovariectomy method, and the OVXDF group was given 0.48g/kg Rhizoma Drynariae solution by gavage for 12 weeks. After 12 weeks, femurs of rats selected for this study were examined with a bone mineral density (BMD) test, Micro-CT, ELISABiochemical testing, hematoxylin and eosin (HE) staining, and immunohistochemistry. A certain portion of the bone tissue was studied with a TMT-based proteomic analysis and functional and pathway enrichment analysis. Finally, key target genes were selected for Western blotting for validation.

**Results:**

The comparison of the OVXDF and OVX groups indicated that *Drynaria rhizome* could improve bone density. In the TMT-based proteomic analysis, the comparison of these two groups revealed a total of 126 differentially expressed proteins (DEPs), of which 62 were upregulated and 64 were downregulated. Further, by comparing the differential genes between the OVXDF and OVX groups and between the OVX and SHAM groups, we concluded that the 27 differential genes were significantly changed in the rats selected for the osteoporosis model after *Drynaria rhizome* intragastric administration. The gene ontology (GO) enrichment analysis of DEPs showed that molecular function was mainly involved in biological processes, such as glucose metabolism, carbohydrate metabolism, immune responses, and aging. A Kyoto Encyclopedia of Genes and Genomes (KEGG) enrichment analysis of DEPs revealed that multiple differential genes were enriched in the estrogen and peroxisome proliferator-activated receptor (PPAR) signaling pathways. Relationships with nitrogen metabolism, glycerophospholipid metabolism, secretion systems, and tumor diseases were also observed. Western blotting was consistent with the analysis.

**Conclusions:**

We used TMT-based proteomics to analyze the positive effects of TCM *Drynaria rhizome*, which can regulate related proteins through the unique roles of multiple mechanisms, targets, and pathways. This treatment approach can regulate oxidative stress, improve lipid metabolism, reduce the inflammatory response mechanism, and improve bone density. These benefits highlight the unique advantages of TCM in the treatment of primary osteoporosis.

## 1 INTRODUCTION

Osteoporosis [1] is a common bone disorder characterized by low bone mass, microstructural destruction of bone tissue, and decreased bone mass [2]. It is estimated that 50%of women and 20% of men over age 50 are at risk of a fracture. Moreover, it has been suggested that the number of people with osteoporosis will double over the next 20 years. The disability rate and case fatality rate create a huge medical burden in today’s society [3]. In clinical diagnosis, bone mineral density (BMD) is measured by dual-energy X-ray absorptiometry [4]. The World Health Organization (WHO) defined osteoporosis as a T-score of –2.5 standard deviations (SD) below the BMD of a healthy younger person of the same sex.

Postmenopausal osteoporosis is caused by an estrogen deficiency, which can lead to degenerative bone loss [5]. The lack of estrogen may result in increased bone resorption, reduced bone formation, and a sharp loss of bone mass in women. In the current treatment of postmenopausal osteoporosis, the most used pharmacotherapy is bone resorption inhibitors, including bisphosphonates, calcitonin, and estrogen receptor modulators [6]. Bone mineralization promoters, such as parathyroid hormone, strontium salt preparations, vitamin K2, and mineralizing drugs (*e.g*., calcium and vitamin D activators) [7], in addition tonifying kidney therapy and bone strengthening through traditional Chinese medicine (TCM), such as treatment with *Drynaria rhizome* or *epimedium*, are also used. *Rhizoma Drynariae* is a commonly used Chinese herbal medicine for the treatment of bone fractures. The main chemical components include flavonoids, triterpenoids, phenylacetones, and lignans [8]. Previous studies have confirmed the positive role of *Drynaria rhizome* in improving bone-promoting fractures. The study by Hu [9] demonstrated that *Rhizoma Drynariae* could improve bone loss caused by regulating the WNT/-catenin pathway in ovariectomized rats in the osteoporosis model. A study [10] showed that *Rhizoma Drynariae* significantly increased antioxidant capacity and BMD and reduced bone mineral loss in osteoporotic rats. The study by Li [11] proved that an *Rhizoma Drynariae* extract can promote the proliferation and osteogenic differentiation of mesenchymal stem cells (MSCs) through various signaling pathways.

In 1994, Marc Wilkins, an American scientist at the University of New South Wales in Australia, first proposed the concept of proteomics. Proteomics is the study of how different proteins interact with each other and the role they play in living organisms through various technical analysis methods, essentially referring to the study of protein characteristics at a large-scale level, including protein expression levels, post-translational modifications, protein-protein interactions, *etc*. [12].

In the current study, it was increasingly confirmed that proteomics is applicable to the basic research and clinical diagnoses of human diseases. It has accurate and comprehensive characteristics for discovering the detailed functional characterization of the disease process and drug therapy [13]. Proteins are essential for all biological processes and are involved in the biological information from all autogenomes [14]. The development of proteomics has important implications for the discovery of diagnostic markers of disease, screening of drug targets, and toxicology studies. In this study, we used a proteomics analysis of the specific targets and pathways of herbal *Drynariae Rhizoma* in ovariectomized rats, thus providing powerful evidence for herbal osteoporosis treatment and a strong reference for clinical medication. Fig. (**1**) shows the specific process flow.

## 2 MATERIALS AND METHODS

### 2.1 Animal Experimental Design

Six-week-old female Sprague-Dawley rats weighing 200–220 g were purchased from Beijing Vital River Laboratory Animal Technology Co., Ltd. Animals were maintained at the SPF Class Animal Experimental Center of Shandong Provincial Hospital of Traditional Chinese Medicine. The feeding environment was characterized by constant humidity (50%) and temperature (25 °C), with indoor lighting for 12 h per day and a standard diet. Experimental animals were bred for 7 d, randomly divided into the experimental group (OVXDF), model group (OVX), and sham surgery group (SHAM). In the OVXDF and OVX groups, a preoperative diet was banned for 12 h. Intravenous pentobarbital sodium (10 g/L) was injected in the Rats, bilateral ovaries were surgically removed from the experimental group (OVXDF), and the model group (OVX), and only small peripheral adipose tissue was removed in the SHAM group. Adaptive rearing was conducted for eight weeks following surgery. After successfully molded, the OVXDF group will refer to the animal dose conversion formula [15], and the *Drynaria rhizome* decoction was administered (intragastric administration) at the gavage dose was 0.48g/kg.. A 0.9% saline solution was administered to the OVX and SHAM groups twice daily (3.0 mL/only) for 12 weeks.

During the preparation of the pharmaceutical solution, we put 100 g of the Chinese medicine *Drynaria rhizome* into a pot, soaked it in water for 1 h, boiled it for 30 min, filtered out the liquid, added water again, 20 min, filtered out the liquid again, fried, two fry mixture, poured it into the beaker, and warmed the fire (100 mL) to obtain the experimental *Drynaria rhizome* water fried liquid (concentration of 1 g/ mL).

Experimental procedures were conducted according to the management and use of experimental animals in Shandong Provincial Hospital of Traditional Chinese Medicine. The operation of the study followed the ethical approval of the Animal Research Ethics Committee, Shandong Provincial Hospital of Traditional Chinese Medicine (affiliate hospital of Shandong University of Traditional Chinese Medicine 2021–26).

### 2.2 Serum Biochemical Indices

Each group of rats was anesthetized with 2% pentobarbital sodium at a dose of 0.2 ml/100 g after 12 weeks [16]. We took blood from the abdominal aortas of the rats in each group, let it stand for 30 min, centrifuged and took the supernatant according to the ELISA kit instructions for osteoprotegerin (OPG) and BMP-2, respectively. According to this operation, the optical density values of each well were detected at a wavelength of 450 nm and substituted into a standard curve to calculate the expression level.

### 2.3 Assessment of BMD and Bone Micro-architecture

The rats in each group died naturally due to blood loss after abdominal aortic blood collection, and the femurs of three rats were taken randomly from each group and stored with paraformaldehyde. Indicators to obtain microstructural characteristics of each group of femurs included BMD, specific bone surface (BS/BV), and bone volume density (BV/TV). The trabecular number (Tb.N) was obtained and analyzed using Micro-CT (SkyScan 1276, German Bruker). Raw images were reconstructed using the 3-D reconstruction software NRecon (software version V1.7.4.2, Bruker, Germany).

#### 2.3.1 Hematoxylin and Eosin (HE) Staining

The left tibias of the rats were placed in a 4% paraformaldehyde fixation solution for 24 h, then in an EDTA decalcification solution at room temperature for 10 d. The decalcification solution was replaced once every other day, and conventional gradient alcohol dehydration was employed, as was paraffin embedding. We made 4 μm, and the slices were placed in a 60 °C oven to dry. The temperature was 4 °C for preservation. We removed the slices and baked them at 60 °C. More than 2 h, avoid dropping. The staining procedure involved conventional xylene dewaxing, gradient alcohol hydration, immersion in a helignolin dyeing solution for 1 min, followed quickly by a tap water rinse until colorless, 1% hydrochloric acid alcohol differentiation for 10 s, and automatic water rinse until colorless. Then, the bones were soaked for 20 min, with the microscopic observation of hematoxylin staining showing that the nucleus was bluish-purple and the interstitium was unstained. A red stain was applied for 1 min, the gradient alcohol was dehydrated, and the neutral gum was sealed. Observation of rat bone histopathology was performed under a microscope.

### 2.4 Immunohistochemistry

In strict accordance with the kit instructions, we added the primary antibody BMP-2 (1:200), Osterix (1:200), and the secondary antibody biotinylated goat anti-rabbit IgG (1:200) and performed optical microscope observation and picture collection. Cytoplasm was stained brown or brown positive. Image-Pro Plus 6.0 image analysis software was used for the analysis.

### 2.5 Tandem Mass Tag (TMT)-labeled Quantitative Proteomics

#### 2.5.1 Protein Sample Preparation

The proteome identification process involved dissolving the protease into peptides, using mass spectrometry, and then deducing the possible proteins. Rat tibias in each group were used for tandem mass tag (TMT) quantitative proteomics testing. After homogenization of the bone tissue, 1000 L of the working solution was mixed with the RIPA lysate and protease inhibitor and then lysed on ice for 5 min. The resulting supernatant was measured at 14,000 rpm/min, 4 °C, and after centrifugation for 15 min.

#### 2.5.2 TMT Labeling

The TMT reagent was removed and thawed at room temperature, and 41 l of acetonitrile was added. Concussion for 5 min, centrifugation. The TMT reagent was added to 100 g of digested samples, and the reaction was performed at room temperature for 1 h. Water with ammonia was added to terminate the reaction. Mixed labeled samples were vortexed and centrifuged to the tube bottom and dried by vacuum freeze centrifugation.

#### 2.5.3 High pH Reversed-phase Fractionation

Mixed labeled samples were dissolved in 100 l of working fluid and centrifuged (14,000 g for 20 min). The supernatant was removed, and the graded gradient was treated in the high-performance liquid phase with a flow rate of 0.7 ml/min.

#### 2.5.4 LC-MS/MS Analysis

The mobile phase A solution (100% water, 0.1% formic acid) and B solution (80% acetonitrile, 0.1% formic acid) were prepared. Lyophilized powder was dissolved using a 10 L A solution and centrifuged (14,000 g for 20 min at 4°C), with the supernatant and tested for liquid quality. Liquid chromatographic elution conditions are shown in Table **1**. We employed the FAIMS Pro™ Interface using an Orbitrap Exploris™ 480 mass spectrometer, Nanospray Flex™ ion source, compensation voltage switches between -45 and -65 every 1 s, and mass spectrometry using a data-dependent acquisition mode. The full mass spectrum scan range was m/z 350–1500. The first-stage mass spectral resolution was set at 60,000 (200 m/z), with AGC for the standard; C-P, the maximum injection time of the trap was Auto. Secondary MS was detected using the “Top Speed” mode. The AGC was set at 100%, and the maximum injection time was on Auto. The peptide fragmentation collision energy was set at 36%, thus generating mass spectral detection of the raw data.

#### 2.5.5 Proteomics Data Analysis

Database selection was based on the required species, database annotation completeness, and sequence reliability. When selecting the database, the following principles were followed. If sequenced organisms, the species database was selected directly, and if non-sequenced organisms were selected, the large proteome database most related to the tested samples was selected.Use this database: Rattus norvegicus database. PD2.4 software was set as follows: the Enzyme was Trypsin; Static Modification was Carbamidomethyl (C); Value of Dynamic Modification was M Oxidation (15.995 Da), TMT-10plex (K, N-terminal), Acetyl (Protein N-terminal); the Value of Precursor ion mass tolerance was ± 15 ppm; the Value of Fragment ion mass tolerance was ± 0.02 Da; The Value of Max Missed Cleavages2.

### 2.6 Bioinformatics Analysis

UniProt was used as the reference database for the rat proteome. Differential genes were selected from the quantitative results and treated as follows. Hierarchical clustering and a global heat map were used to exhibit the DEPs. DAVID Bioinformatics Resources v6.8 (https://david.ncifcrf.gov/) was used to analyze gene ontology (GO: contains biological processes (BPs), cellular components (CCs), molecular function (MF), and Kyoto Encyclopedia of Genes and Genomes (KEGG) pathways). STRING 11.0 (http://www.string-db.org/) was used to create protein-protein interaction (PPI) networks.

### 2.7 Western Blotting

Bone protein extraction was performed at 4 °C according to the instructions(Minute™ Total Protein Extraction Kit for Bone Tissue,SA-02-BT). The sample was fully soaked with pH 7.4 PBS twice, and bone tissue was fragmented and weighed (mass). The bone protein extract (200 mg of bone tissue to protein extract 500 L) was added and homogenized with a homogenizer. The homogenate was shaken at 4 °C for 30 min and centrifuged at 12 000 r min-1 for 10 min. The mixture of protein and loading buffer was boiled at 95 °C. We isolated 10 μL of total protein with 5% and 10% SDS-PAGE and transferred it to a polyvinylidene fluoride (PVDF, 0. 45 μm) membrane. We used 3% bovine blood. After 1 h of albumin blocking, it was combined with, rabbit anti-MMP2 (Matrix Metallopeptidase 2) (1:1000), rabbit anti-PIN1(Peptidylprolyl Cis)(1:1000), rabbit anti-DBI(Diazepam Binding Inhibitor)(1:600), and rabbit anti-RAP1B (Ras-Related Protein Rap-1b)(1:1000) at room temperature. Lower closure with β-actin (1:3000) was the standard. Then, we used the union-suitable secondary antibody treatment. Chemiluminescent HRP substrates detected antigen-antibody complexes. We used the associated Image J for the optical density analysis.

### 2.8 Statistics

SPSS26.0 statistical software was used for statistical analysis. One-way ANOVA was used to compare groups using the LSD test, and the results were expressed as (). *P*<0.05 was considered statistically significant.

## 3 RESULTS

### 3.1 Biochemical Index Detection

The concentrations of BMP-2 and Osterix in the plasma of rats in the experimental and model groups were determined by the ELISA kit. The results showed that BMP-2 and Osterix levels in the OVX group were lower than in the SHAM group and the BMP-2 and Osterix levels in the OVXDF group were significantly increased compared with the OVX group. (*P* < 0.05). These results show that *Rhizoma Drynariae* had a significant therapeutic effect on osteoporosis in rats, as shown in Fig. (**2**).

### 3.2 Assessment of BMD and Bone Micro-architecture

The Micro CT results showed that the femoral bone mass in the OVX group was reduced, and the femoral trabecular structure was damaged and sparse. as shown in Fig. (**3**). Compared with the SHAM group, BMD, BS/BV, BV/TV, and Tb.N were significantly reduced in the OVX group (*P* < 0. 05). The OVXDF group received *Drynaria rhizome* showed significant upregulation and a return to rats’ normal levels (*P* < 0. 05), as shown in Fig. (**4**). The rats in the OVXDF group had dense bone trabeculae and greater bone mass. The results of the Micro-CT bone assessment showed that *Rhizoma Drynariae* has a significant effect on improving the microstructure of bone.

### 3.3 HE Staining

In the OVX group, rat femurs could be seen as thinning and decreasing, with fracture destruction, increased bone resorption holes, and obvious osteoporosis characteristics. Rats in the SHAM group had abundant cancellous bone, a large number of bone trabeculae, a tight arrangement, a complete bone tissue structure, and a thick and uniform wall. The femoral trabecular structure in rats in the OVXDF group was relatively complete. The texture was arranged tightly, the bone trabecular connectivity was better, and the wall thickness was denser. The state of the trabecular bone and cancellous bone was superior to that of the OVX group, and the difference was statistically significant (*P* < 0. 05). The results showed that *Rhizoma Drynariae* had a therapeutic effect on osteoporosis, as shown in Fig. (**5**).

### 3.4 Immunohistochemistry

BMP-2 and Osterix protein expressions appeared brownish-yellow after staining. The staining results showed that the positive area in the SHAM group and the OVXDF group was greater than that in the OVX group. The statistical results show that the expression of the OVXDF group was higher than that of the OVX group, and the difference was statistically significant (*P* < 0. 05), as shown in Fig. (**6**).

### 3.5 TMT Quantitative Proteomics of the DEPs Between Two Groups

The TMT proteomics identification results were evaluated from two levels: peptide and protein. Combined with the differences (FOLDCHANGE > 1.2, 0.83)and *P* < 0.05, a total of 347,739 spectra were analyzed with the screening test of protein markers (matching spectra: 41,881, peptides: 29,979). The number of proteins identified was 3,424. (Note the condition with fold > 1.2 or < 0.8 and *P* < 0.050. The DEPs were analyzed further. Accordingly, 126 DEPs were found in the OVXDF/OVX groups, of which 62 were upregulated, and 64 were downregulated, as shown in Fig. (**6**). We compared the differential genes between the OVXDF/ OVX groups and between the OVX/ SHAM groups. We concluded that the following 27 different genes were significantly changed in the rats used in the osteoporosis model after *Drynaria rhizome* intragastric administration: PLTP, PON1, NDUFA5, SLC4A1, TNPO1, RAP1B, A1M, TFRC, AFM, PARP14, CRIP1, CZIB, CCDC25, MMP2, LGALS1, FABP5, PIN1, PDGFRL ST13, LIMA1, IKBIP, SORBS1, SLC9A3R2, PPP1R12B, ERC1, DBI, NQO2 (Table **1**). From the KEGG enrichment analysis, the following differential genes with osteoporosis were more related: PLTP, PON1, RAP1B, TFRC, MMP2, LGALS1, PIN1, PDGFRL, and SORBS1.

### 3.6 Enrichment of GO Analysis

Gene ontology (GO) describes the function, location, and activity of genes, divided into three subcatalogues: CC, MF, and BP. Results of the DEP enrichment analysis showed that the CC was mostly concentrated in the nucleus, cell membrane, and cytoplasm, as well as in the organelles, such as the mitochondria and Golgi apparatus. Participation in MF mainly enriched metal ions, receptors, rRNA, protein, integrin binding and signal sensor activity, and transmembrane transporter activity, whereas BP mainly enriched cell signal transduction, transmembrane transport, protein transport and guidance, cell division proliferation movement, cell adhesion, cell junction organization involving glucose and carbohydrate metabolism, the immune system, and aging as shown in Fig. (**7**).

### 3.7 Enrichment of the KEGG Pathway Analysis

The KEGG analysis of DEPs found that such DEPs as Src, RAP1B, Rlc-a, Rap1a, Ca2, CFL2, MMP2, ATP1B1, HSP90AA1, and PIP4K2B were significantly enriched, and differential genes were mostly enriched in the upregulated estrogen signaling and downregulated PPAR signaling pathways. Nitrogen metabolism, glycerophospholipid metabolism, pancreatic secretions, collected duct acid secretions, homologous recombination, and platelet activation upregulated. Bile secretions, proximal tubule bicarbonate reclamation, aldosterone-regulated sodium reabsorption, fluid shear stress, and atherosclerosis, as well as ubiquinone and terpenoid-quinone biosynthesis performance, downregulated. Further, results regarding DEPs showed that bladder cancer and Cushing syndrome had strong correlations. Leukocyte transendothelial migration also showed a strong enrichment relationship in the regulation of actin cytoskeletonas shown in Fig. (**8**).

### 3.8 PPI Network Analysis

We used the STRING database to determine the relationship among DEPs. Of the 126 identified DEPs, each node represented a DEP, and the lines between the nodes indicated an interaction between the two proteins. Furthermore, 97 of the identified DEPS were interrelated. The PPI network had 125 nodes and 148 edges connected to each other. The average number of nodes was 2.89, and 97 DEPs were interconnected, as shown in Fig. (**9**).

### 3.9 Western Blotting

We randomly selected the top-ranking proteins MMP2, PIN1, DBI, and RAP1B to perform WB with the aim of verifying the reliability of the proteomic results of TMT. The expression level of PIN1 significantly down regulation in the femurs of rats and DBI in the OVX group compared to the SHAM group (*p* < 0.05). After treatment with *Drynaria rhizome*, the expression levels of MMP2,, DBI, and RAP1B proteins significantly returned to the levels considered normal for rats (*p* < 0.05). These results are consistent with the protein-based proteomics analysis of TMT, as shown in Fig. (**10**).

## 4 DISCUSSION

Osteoporosis (OP) is a systemic metabolic bone disease characterized by osteopenia, changes in bone microstructure, accompanied by increased fragility of bone and easy fracture, and its occurrence and development is a multifactorial, multi-gene participation, multi-stage extremely complex biological process [17]. Drugs for the treatment of osteoporosis in the clinic can be roughly divided into bone resorption inhibitors to prevent bone loss and bone formation stimulants that promote bone formation, so drug treatment is a comprehensive treatment, and then supplement calcium while supplementing other preparations. to achieve an increase in bone mass [18].

We compared the ovariectomy-induced osteoporosis model rats with the sham surgery group and the drug treatment group to analyze and observe the differences in bone quality and indicators among the groups.OPG is an inhibitor that can suppress bone destruction during bone resorption. It prevents the binding of RANKL (receptor activator of nuclear factor kappa-B ligand) to RANK (receptor activator of nuclear factor kappa-B) by binding to RANKL. RANKL is a factor responsible for stimulating bone resorption, as it promotes the formation and activation of osteoclasts, which are cells that break down bone. If the level of OPG is high, it can compete with RANKL to bind to RANK, thereby blocking the RANKL-RANK signaling pathway, reducing the formation of destructive osteoclasts, and decreasing the degree of bone resorption. Therefore, OPG is closely associated with the extent of bone destruction and can serve as an indicator of bone health.In summary, OPG can reflect the degree of bone destruction, and BMP-2 can reflect the ability of bone formation. Therefore, these two proteins can be used together as indicators to evaluate bone health.In contrast to ovariectomy(OVX), surgical removal of the fat above the ovaries does not affect bone quality in rats. This is because bone health is mainly regulated by hormones, and the ovaries are primarily responsible for estrogen secretion, which plays a crucial role in bone density and health. Relative ovariectomy only removes the ovaries without affecting other endocrine organs and hormone secretion, so the bone quality in rats remains normal. On the other hand, surgical removal of the fat above the ovaries only removes some fat tissue and does not directly impact the endocrine system or bone health.

At present, traditional Chinese medicine, with its unique therapeutic advantages in clinical practice more and more application and attention. A large number of ancient documents record that China began to use a variety of bone-strengthening traditional Chinese medicine in ancient times to treat various orthopedic diseases, and the effect is very significant. Many types of traditional Chinese medicines can have a positive therapeutic and protective effect on bones, among which bone fragmentation is more commonly used. Rhizoma Drynariae as a plant. We generally select its roots to treat bone diseases. Modern pharmacological research [19] has shown Rhizoma Drynariae can promote BMSCs proliferation and osteogenic differentiation and significantly improve ovarian-induced bone loss and can also be activated by activating the Wnt/β-catenin pathway [19]. Multiple mechanisms, such as inhibiting PPARγ pathway, promoting osteogenic differentiation of BMSCs, inhibiting their adipogenic differentiation and maturation, regulating OPG/RANKL ratio, and indirectly inhibiting osteoclast. However, the specific mechanism and target of Rhizoma Drynariae for the treatment of postmenopausal osteoporosis are still complex, so based on the above reasons, we further apply proteomics technology based on previous research for further analysis. We used a TMT-based proteomic analysis to analyze the intervention results of TCM *Drynaria rhizome* in ovariectomized rats and revealed the specific mechanism and target of the TCM use of this herb in the treatment of postmenopausal osteoporosis. In this analysis, we obtained 126 DEPs by comparing the experimental and model groups. We further compared the differential genes between the OVXDF and OVX groups and between the OVX and SHAM groups in the osteoporosis model rats after treatment (*i.e*., PLTP, PON1, NDUFA5, SLC4A1, TNPO1, RAP1B, A1M, TFRC, AFM, PARP14, CRIP1, CZIB, CCDC25, MMP2, LGALS1, FABP5, PIN1, PDGFRL, ST13, LIMA1, IKBIP, SORBS1, SLC9A3R2, PPP1R12B, ERC1, DBI, NQO2).

After performing the enrichment analysis with Differential Genes, we found that TCM *Drynaria rhizome* could act on mitochondria and other organelles to regulate RNA, as well as protein receptors and signaling, thus playing an active role in fighting oxidative stress. Meanwhile, in the related signaling pathways, the positive effect of *Drynaria rhizome* also had a strong correlation with estrogen, lipid metabolism, and the digestive system. Estrogens are steroid hormones that regulate a plethora of physiological processes in mammals, including reproduction, cardiovascular protection, bone integrity, cellular homeostasis, and behavior. The estrogen signaling pathway is able to exert antioxidant effects and inhibit oxidative stress [20]. It is also required for bone tissue formation, and we know that an estrogen deficiency also leads to increased osteoclastogenesis and enhanced bone resorption. In menopausal women, a sharp loss of pine and cortex bone occurs due to an estrogen deficiency in the body [21]. Increased bone resorption and decreased bone formation lead to reduced bone mass, structural disturbances, and reduced bone strength [22]. Oestrogen has been used as one of the treatment options for women with postmenopausal osteoporosis [23].

This analysis verified that TCM *drynaria rhizome* could upregulate the estrogen system, which can specifically reverse osteoclastic differentiation, enhance osteogenesis, and improve osteoporosis, This finding is consistent with Wang’s experimental study [24]. Our enrichment analysis confirmed that Estrogen is more related to aging than cardiovascular disease, Alzheimer’s disease, Parkinson’s disease, *etc.* [25]. Among them, the expression of LGALS1 gene is more prominent.LGALS1 is currently found to be involved in the regulation of the bone marrow microenvironment and related to aging. It is a key immunosuppressive molecule that is mostly found in cancer. Previous studies confirmed substantial expression in BMSC and involvement in the regulation of BMSC differentiation and bone homeostasis. This suggests that LGALS1 may be an important predictor of the progression of osteoporosis [26]. 82 male volunteers were included in one study [26] in which they were tested for BMD and serum LGALS1 levels. The results showed a positive correlation between human BMD and serum LGALS1 levels. A comparison of mRNA in bone expression in individuals with proximal femoral neck fractures (OP) or osteoarthritis with individuals without such issues, as well as a comparison with other genes, including LGALS1, verified less expression in OP patients [27].

In recent years, studies have shown that abnormal mineral metabolism, osteopenia/osteoporosis, and even fragility fractures are common in animal models of primary aldosteronism and hyperaldosteronism and in patients with these conditions [28]. Findings from this study showed that *Drynaria rhizome* fostered the downregulation of sodium reabsorption, which confirmed the mechanism of the aldosterone system in osteoporosis and the beneficial regulatory effect of *Drynaria rhizome*. In the protein analysis of the experimental and model groups, we detected multiple target proteins of the PPAR signaling pathway (PLTP, FABP5, SORBS1, and DBI). Previous studies [29] have demonstrated the importance of PPAR signaling pathway conduction in the differentiation of MSCs into adipocytes and osteoblasts. Peroxisome proliferator-activated receptors (PPARs) are nuclear hormone receptors that are activated by fatty acids and their derivatives [30, 31]. Previous studies have confirmed that targeted inhibition of the PPAR-pathway can inhibit oxidative stress and effectively reverse osteoporosis. At present, PPAR mostly regulates it in obesity and its triggered inflammation [32]. It is also involved in the entire process of lipid metabolism, including adipocyte development, lipoprotein metabolism, and glucose homeostasis [33]. We know that in the bone marrow, stem cells can be differentiated into osteoblasts and adipocytes, and the pathogenesis of osteoporosis for bone formation and bone marrow fat increases. Bone marrow (MSCs) exhibits osteogenic differentiation and adipogenic differentiation ability, contributing to the development of osteoporosis to a certain extent [34, 35]. Previous experimental studies [36] confirmed that the adiidation mediated by PPAR signaling in ovariectomized rats, further verifying the regulatory effect of *Drynaria rhizome* on PPAR signaling, which can upregulate PON1, SORBS1, and other contributing bone genes. Further, it can downregulate FABP5 and DBI, contributing to lipid gene expression. Clinical data suggest that oxidative stress is associated with bone resorption because it causes the loss of BMD [37]. PON1 is one of the regulatory indicators of peroxidase-like activity with the ability to inhibit lipid peroxidation as an antioxidant [38]. Current studies show that although lipid oxidation products can improve the calcification of vascular cells because they hinder the osteogenic transformation of osteocytes [39]. Multiple studies have demonstrated that PON1 may be an important gene in the pathogenesis of postmenopausal osteoporosis. PON1 is able to regulate high-density lipoprotein to enhance antioxidant properties and reduce the accumulation of lipid peroxidation products [40]. It improves bone mass by enhancing oxidative stress. Osteoporosis patients showed reduced serum PON1 activity when compared with healthy individuals [41, 42]. PON1 was able to significantly reduce osteoblast apoptosis and improve bone turnover markers, such as BMP-2 [43].

Previous studies [44] have confirmed that primary osteoporosis has a strong correlation with a variety of inflammatory and immune systems. After we analyzed *Drynaria rhizome* intervention and ovarian osteoporosis model rats, we found that multiple inflammatory immune signal factors and pathways were regulated. Some inflammatory signaling pathways were inhibited, while endocrine and other pathways were positively regulated. Among them, the PDGFRL with a large downregulation difference belonged to the platelet-derived growth factor receptor. Mutations in this gene, or deletion of a chromosomal segment containing the gene, were associated with sporadic hepatocellular carcinomas, colorectal cancers, and non-small cell lung cancers. This finding suggests that this gene product may function as a tumor suppressor [45, 46]. *In vitro* evidence for the role of PDGF signaling in osteocytes has been established, possibly by promoting OPG production upon inhibiting PDGFR signaling, thus stimulating bone formation [47]. A recent study showed that dexamethasone could affect the activity of the PDGFR signaling pathway in rats and cause osteoporosis in women [48]. In addition, we verified the positive effect of *Drynaria rhizome* in the inflammation of endocrine and, digestive system and immune system inflammations. Numerous studies have shown that patients with pancreatic dysfunction had osteopenia and diagnosed osteoporosis [49, 50]. This may be due to a vitamin D deficiency caused by inflammation, such as pancreatitis, and changes in bone metabolism caused by the imbalance in nutritional status [51]. From the collection of duct acid secretions, inflammation of the digestive system (*e.g*., inflammation of the gastrointestinal tract) is often accompanied by a calcium absorption disorder, which also leads to the loss of BMD. In bile secretions, bile acids are components of bile and play important roles in glucose and lipid metabolism and the regulation of intestinal microbiota. In the current study, bile acids had a positive protective effect on bones [52, 53]. The study by Robert Eriksson *et al*. [54] revealed that BMD increased significantly after administration of the bile acid receptor agonist after an ovariectomy.

At present, many studies have confirmed the positive effect of *Drynaria rhizome* in promoting osteoblast proliferation and inhibiting osteoclast differentiation [55, 56]. Target proteins regulating osteogenesis and osteoclasts were also detected in our experiments. In a recent study, PIN1 [57] was demonstrated in osteoporosis, mainly because the functional interaction between RUNX2 and PIN1 plays a key role in the skeletal system [58]. PIN1 can enhance BMP and WNT signaling involved in the regulation of RUNX2 and Osterix [57, 59]. Experimental data suggest that inhibition of PIN1 mediates RUNX2 phosphorylation and is able to attenuate RUNX2 protein levels, affecting osteogenic differentiation. The data suggest that PIN1 plays a role in osteoclast formation and bone resorption by regulating the osteoclast fusion protein DC-STAMP. Osteogenic cell LRP1 was identified as a key gene in mouse bone mass. The study by Alexander Bartelt [60] revealed LRP1-mediated regulation of PDGFRβ signaling through RANKL production *in vitro* and *in vivo*. Previous studies [61] have investigated the pharmacological effect of *Drynaria rhizome* total flavonoids (TFRD) on type H blood vessels. TFRD not only enhanced the osteogenic activity of BMSC, but it also increased the abundance of type H blood vessels, showing better angiogenic and osteogenic outcomes. This finding is similar to the results of the differential gene analysis, increasing the credibility of the validation results. Genetic analyses suggest that RAP1B is essential for the development of the craniofacial and body bones and that RAP1B inhibits osteoblast differentiation by inhibiting MAPK signaling [62]. RAP1B is related to the Ras-associated protein 1 and is positively correlated with osteoclast differentiation. In this study, down-regulation of RAP1B inhibited osteoclast differentiation [62, 63].

In this analysis, MMP2 in the OVXDF group and rats in the OVX group were significantly regulated, and the results showed that MMP2 and osteoporosis were inextricably linked. MMP2 is expressed as an MMP family member in osteoblasts and osteoclasts [64]. It is able to promote osteoclast migration to the bone surface, and changes in MMP2 affect the balance between osteogenic formation and bone resorption [65, 66]. In Zheng’s study [67], the expression levels of MMP2, mRNA, and protein increased faster between 20 and 24 weeks after surgery when compared with SHAM, suggesting that decreased estrogen levels caused a decrease in MMP2 inhibition and consequent bone destruction. In addition to confirming the mechanism of the action of MMP2 in primary osteoporosis, the SUN study [68] confirmed its similar involvement in the pathological process of glucocorticoid-induced osteoporosis. In mice in a prednisolone-induced osteoporosis model, a histochemical analysis of the tibia revealed a prednisolone-induced increased expression in MMP2, 9, and 13 in the bone tissue of mice. Previous clinical studies showed that serum MMP2 concentrations were significantly higher in postmenopausal women with osteoporosis than in normal controls [69]. It is, therefore, reasonable to infer that MMP2 could be a therapeutic target for the treatment of osteoporosis.SORBS1 refers to the insulin signaling molecules expressed in fat and skeletal muscle that is involved in the regulation of signaling pathways within the cytoskeleton [70, 71]. XU *et al*. [72] demonstrated the positive regulation of SORBS1 in osteogenic differentiation. PLTP is a member of the lipid transfer/LPS binding protein gene family with unique functions in remodeling high-density lipoprotein, which has important roles in lipid metabolism and inflammatory immunity. It is highly expressed in the plasma of patients with type 2 diabetes and obesity [73]. Experiments regarding the hMADS cell culture and induction have shown that PLTP secretions increased during adipocyte differentiation [74]. Therefore, based on the experiments, the improvement of osteoporosis improved by *Rhizoma Drynariae* may result from the ability to upregulate osteogenesis-related genes and inhibit the expression of osteocast and adipogenic-related genes, which participating in the regulatory mechanism. Currently, the association between ferroptosis and postmenopausal osteoporosis is widely studied [75]. It is worth noting that in the analysis of differential genes, we found that the standard proteins of ferroptosis TFRC were significantly regulated after *Rhizome Drynaria* intervention, so we can speculate that ferroptosis also plays an important role in osteoporosis. Therefore, the intervention mechanism and key targets of ferroptosis in osteoporosis may be the next research focus of our group.

We performed an enrichment analysis of differentially expressed proteins in the OVX and OVXDF. Subsequently, we constructed a protein-protein interaction network between the two groups. However, we found that some proteins in the network had poor correlation with each other. We believe that there might be several reasons for this observation. Firstly, the PPI network was constructed based on available information from public databases, which may not accurately reflect the true interactions between these proteins *in vivo*. Secondly, the differential expression of some proteins between the two groups may have been influenced by non-specific factors, such as individual variations or technical errors. To address this issue, we plan to include more experimental data in future studies to validate the interactions and also to perform statistical analysis to identify the most significant interactions in the network. We appreciate your insightful comments and will consider your suggestions in our future work.

## CONCLUSION

In summary, we used a TMT-based proteomics analysis of inflammatory *Drynaria rhizome* to target and regulate PLTP, PON1, RAP1B, TFRC, MMP2, LGALS1, PIN1, PDGFRL SORBS1 and other related proteins. The estrogen signaling pathway, PPAR signaling pathway, erroptosis, and inflammatory immune system played roles in regulating hormone levels. We assessed resistance to oxidative stress, improvements in lipid metabolism, a reduction in the inflammatory mechanisms, and the effect on osteogenic, adipogenic, and osteoclastic differentiation and other aspects of the positive improvements in bones. In this study, we highlighted the multiple mechanisms of TCM treatment for primary osteoporosis and the unique action characteristics of multiple targets and pathways.

## Figures and Tables

**Fig. (1):**
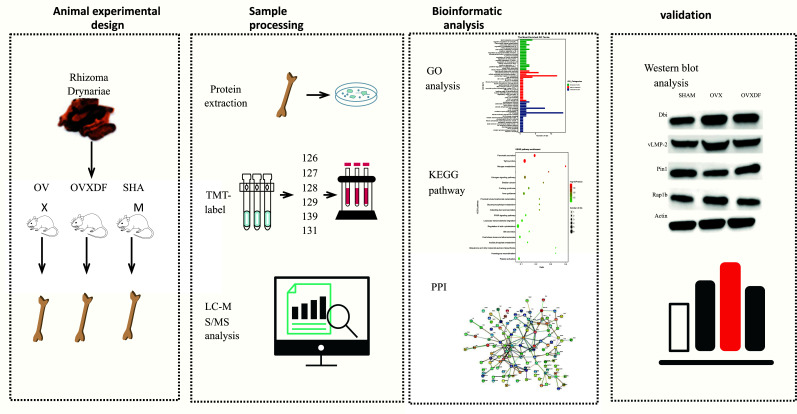
Process of TMT Quantitative proteomics analysis of rat bone tissue proteins.

**Fig. (2):**
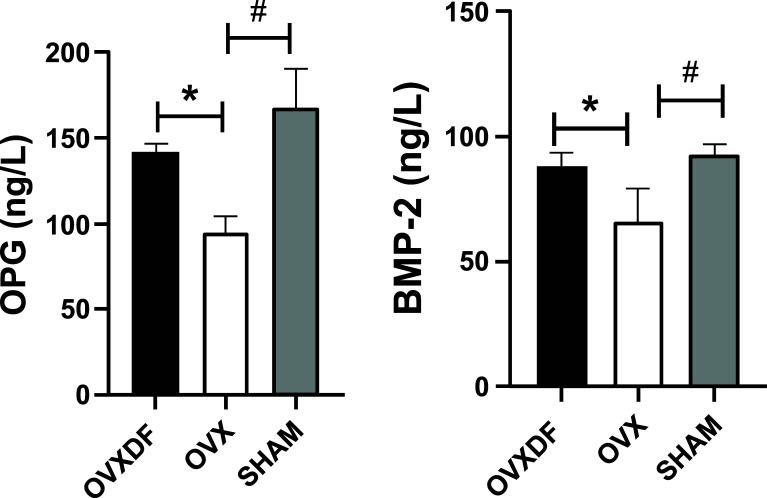
Serum OPG and BMP-2 concentrations comparison among three groups rats, compared with SHAM group, #*P*<0.05. compared with OVX group, *P<0.05.

**Fig. (3):**
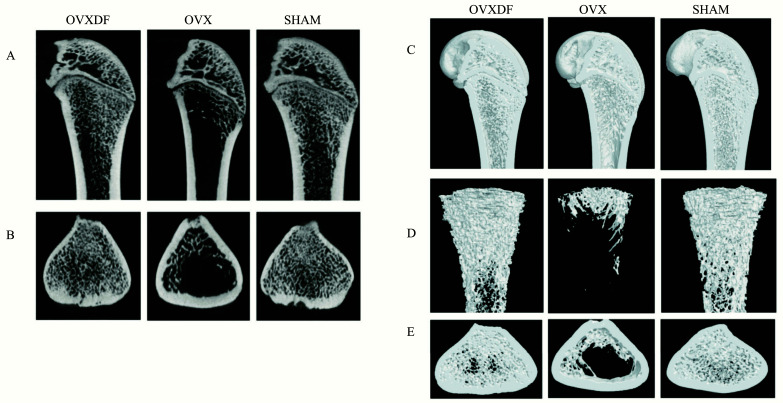
Micro-CT images of the femur. (**A** and **B**) 2D images in three groups of the distal femur (scale bars, 1 mm). (**C**-**E**) 3D images in three groups The proximal femur (scale bars, 1 mm).

**Fig. (4):**
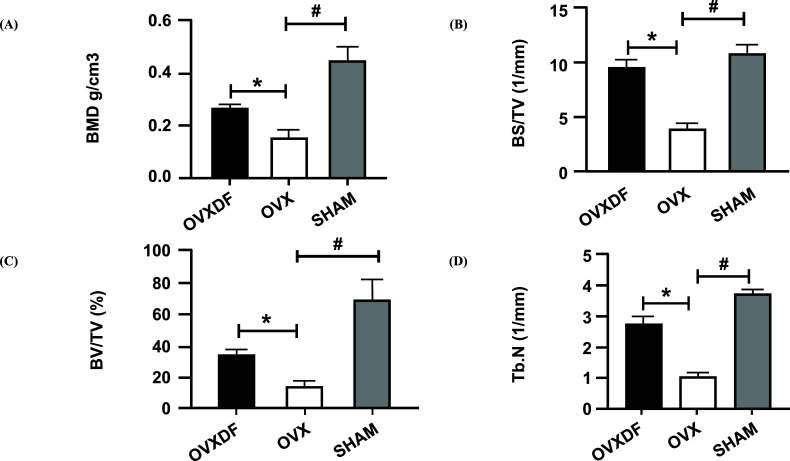
Bone parameter values of Micro-CT (**A**). The BMD indicates the bone mineral density in the bone tissue of the region of interest. (**B**) Bone surface area and tissue volume ratio (BS / TV) can indirectly reflect the amount of bone mass (**C**). Bone body and fraction (BV / TV) are commonly used in the evaluation of cortical and cancellous bone mass, (**D**) Number of trabecular bone (Tb.N) to evaluate the spatial morphological structure of trabecular bone. # *P* <0.05 *versus* sham group, * *P* <0.05, versus model groups, n = 3 per group, Data were shown as mean ± SD.

**Fig. (5):**
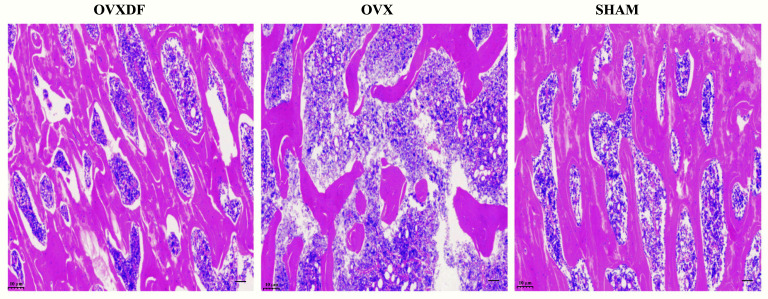
(**A**) H&E staining images of femur bone (100 um, 100x).

**Fig. (6):**
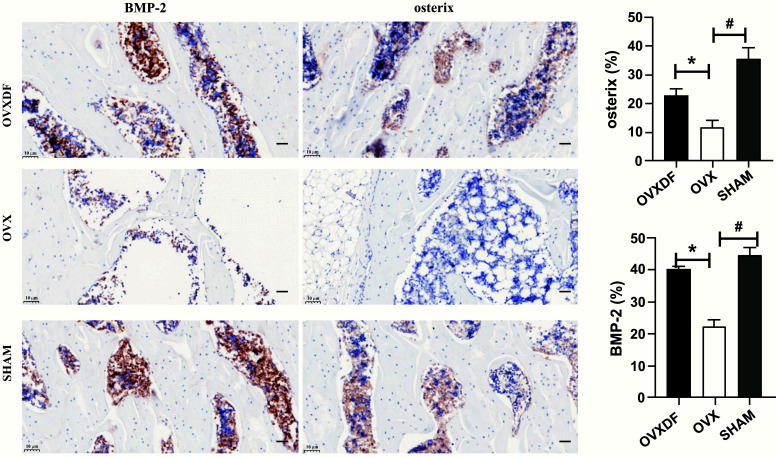
BMP-2 and osterix expression in bone tissue among three groups rats (50um, 400×). compared with SHAM group, #*P*<0.05. compared with OVX group, *P<0.05.

**Fig. (7):**
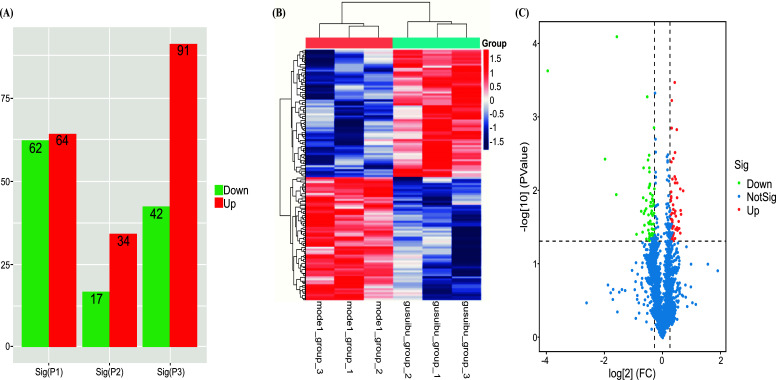
(**A**) Column diagram of DEGs expression analysis between groups, with color representing upregulated and downregulated DEGs. (**B**) Heat map of sample differential protein clustering, gusuibu=OVXDF group. (**C**)Volcano plot of DEGs between the OVXDF group and the OVX group: The two vertical dashed lines in the figure are the threshold of expression difference multiple; the horizontal dashed line is the significance level threshold.Colors indicate that genes are up、 downregulated, or not significantly differentially expressed. Sig(P1) =OVXDF group_ OVX group),Sig(P2) = Sig(OVXDF group_sham group),Sig(P3) = Sig(OVX group_sham group).

**Fig. (8):**
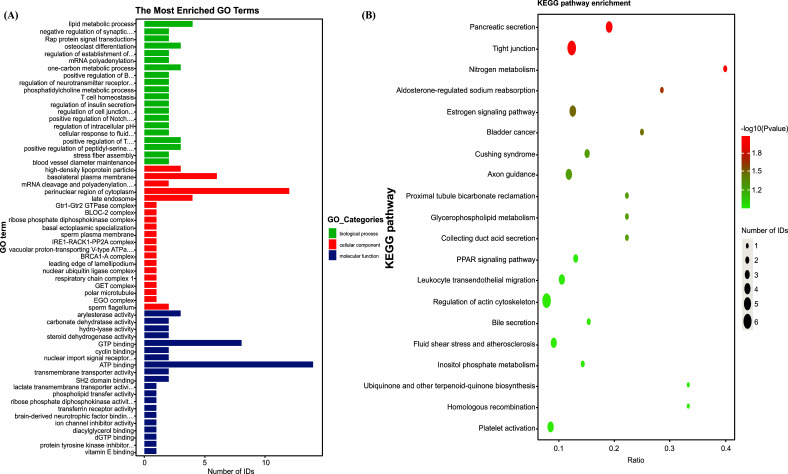
Bioinformatics analysis od DEFs involved in biological processes, (**A**) enriched GO information, (**B**) enriched KEGG information.

**Fig. (9):**
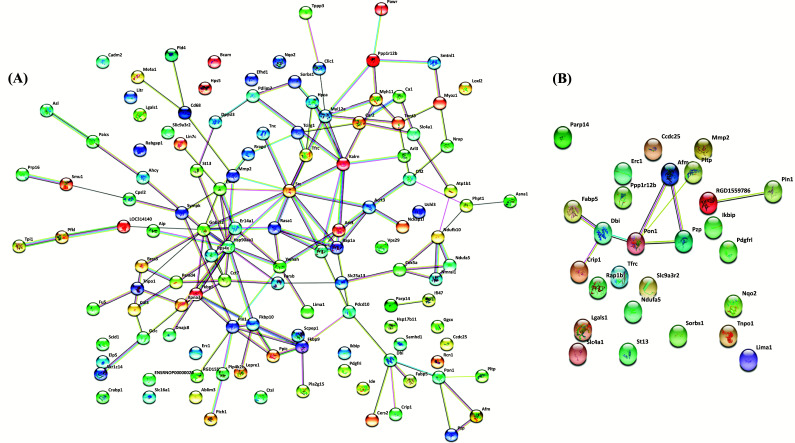
(**A)** PPI network diagram of the DEGs between the OVXDF group and the OVX group. (**B**) PPI network diagram of the 27 DEGs with significant changes after drynaria rhizome treatment.

**Fig. (10):**
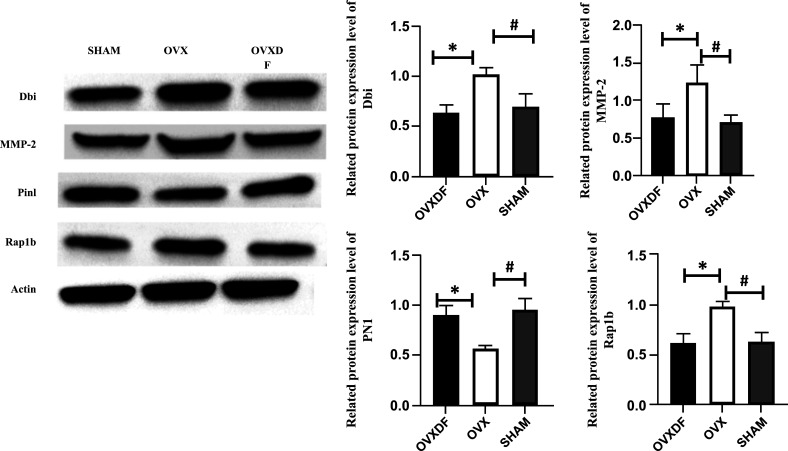
Expression levels of the related proteins. compared with SHAM group, #*P*<0.05. compared with OVX group, *P<0.05.

**Table 1:** 27 significant DEGs .(1:up-regulation, -1:down regulation).

**Gene Name**	**Description**	**OVXDF v OVX**	**OVX v SHAM**
Pltp	Phospholipid transfer protein	-1	1
Pon1	Serum paraoxonase/arylesterase 1	1	-1
Ndufa5	NADH dehydrogenase [ubiquinone] 1 alpha subcomplex subunit 5	1	-1
Slc4a1	Anion exchange protein	1	-1
Tnpo1	Transportin 1	1	-1
Rap1b	Ras-related protein Rap-1b	-1	1
A1m	Alpha-1-macroglobulin	1	-1
Tfrc	Transferrin receptor protein 1	1	-1
Afm	Afamin	1	-1
Parp14	Poly [ADP-ribose] polymerase	1	-1
Crip1	Cysteine-rich protein 1	-1	1
Czib	CXXC motif containing zinc binding protein	-1	1
Ccdc25	Coiled-coil domain-containing protein 25	-1	1
Mmp2	72 kDa type IV collagenase	-1	1
Lgals1	Galectin-1	1	-1
Fabp5	Fatty acid-binding protein 5	-1	1
Pin1	Peptidyl-prolyl cis-trans isomerase	1	-1
Pdgfrl	Platelet-derived growth factor receptor-like protein	-1	1
St13	Hsc70-interacting protein	-1	1
Lima1	LIM domain and actin-binding protein 1	-1	1
Ikbip	Inhibitor of nuclear factor kappa-B kinase-interacting protein	-1	1
Sorbs1	Sorbin and SH3 domain-containing protein 1	1	-1
Slc9a3r2	Na(+)/H(+) exchange regulatory cofactor NHE-RF	-1	1
Ppp1r12b	Protein phosphatase 1 regulatory subunit	-1	1
Erc1	ELKS/Rab6-interacting/CAST family member 1	-1	1
Dbi	Acyl-CoA-binding protein	-1	1
Nqo2	Ribosyldihydronicotinamide dehydrogenase [quinone]	-1	1

## Data Availability

The datasets generated and analyzed during the current study are not publicly available but are available from the corresponding author. The original contributions presented in the study are publicly available. Data are available *via* ProteomeXchange with identifier PXD035745.

## LIST OF ABBREVIATIONS

CT: Computerized Tomography
DEG: Differentially Expressed Gene
ECM: Extracellular Matrix
H&E: Hematoxylin and Eosin
GO: Gene Ontology
KEGG: Kyoto Encyclopedia of Genes and Genomes
PPI: Protein-protein Interaction
STRING: Search Tool for the Retrieval of Interacting Genes
T2DM: Type 2 Diabetes Mellitus

## References

[1] Lane J.M., Russell L., Khan S.N. (2000). Osteoporosis.. Clin. Orthop. Relat. Res..

[2] Coughlan T., Dockery F. (2014). Osteoporosis and fracture risk in older people.. Clin. Med. (Lond.).

[3] Lamichhane A.P. (2005). Osteoporosis-an update.. JNMA J. Nepal Med. Assoc..

[4] (1994). Assessment of fracture risk and its application to screening for postmenopausal osteoporosis. Report of a WHO Study Group.. World Health Organ. Tech. Rep. Ser..

[5] Gambacciani M., Levancini M. (2014). Management of postmenopausal osteoporosis and the prevention of fractures.. Panminerva Med..

[6] Eastell R., Walsh J.S., Watts N.B., Siris E. (2011). Bisphosphonates for postmenopausal osteoporosis.. Bone.

[7] Hurley D.L., Khosla S. (1997). Update on primary osteoporosis.. Mayo Clin. Proc..

[8] Chen S.Q., Liang W., Zhang X.M., Li X., Zhan Z.L., Guo L.P., Huang L.Q., Zhang X.M., Gao W.Y. (2021). Research progress on chemical compositions and pharmacological action of Drynariae Rhizoma.. Zhongguo Zhongyao Zazhi.

[9] Hu Y., Mu P., Ma X., Shi J., Zhong Z., Huang L. (2021). Rhizoma drynariae total flavonoids combined with calcium carbonate ameliorates bone loss in experimentally induced Osteoporosis in rats *via* the regulation of Wnt3a/β-catenin pathway.. J. Orthop. Surg. Res..

[10] Mu P., Hu Y., Ma X., Shi J., Zhong Z., Huang L. (2021). Total flavonoids of Rhizoma Drynariae combined with calcium attenuate osteoporosis by reducing reactive oxygen species generation.. Exp. Ther. Med..

[11] Yang L., Zhu X.F., Wang P.P., Zhang R.H. (2013). Effects of drynariae rhizoma water-extraction on the ability of osteogenic differentiation and it’s mechanism. Zhong Yao Cai.

[12] Mendes M.L., Dittmar G. (2022). Targeted proteomics on its way to discovery.. Proteomics.

[13] Cifani P., Kentsis A. (2017). 2017, Towards comprehensive and quantitative proteomics for diagnosis and therapy of human disease.. Proteomics.

[14] Cheung C.H.Y., Juan H.F. (2017). Quantitative proteomics in lung cancer.. J. Biomed. Sci..

[15] Blanchard O.L., Smoliga J.M. (2015). Translating dosages from animal models to human clinical trials—revisiting body surface area scaling.. FASEB J..

[16] Niamh Clancy (2023). The Veterinary Nurse’s Practical Guide to Small Animal Anaesthesia..

[17] Reid I.R., Billington E.O. (2022). Drug therapy for osteoporosis in older adults.. Lancet.

[18] LeBoff M.S., Greenspan S.L., Insogna K.L., Lewiecki E.M., Saag K.G., Singer A.J., Siris E.S. (2022). The clinician’s guide to prevention and treatment of osteoporosis.. Osteoporos. Int..

[19] Yan L.I. (2019). Effects of bone fragmentation hydration decoction on lipid differentiation of bone medullary stem cells in osteoporotic rats with osteoporosis removal by Wnt/β-catenin pathway.. Chin. J. Trad. Chin. Med. Pharm..

[20] Haas M.J., Raheja P., Jaimungal S., Sheikh-Ali M., Mooradian A.D. (2012). Estrogen-dependent inhibition of dextrose-induced endoplasmic reticulum stress and superoxide generation in endothelial cells.. Free Radic. Biol. Med..

[21] Bolognese M.A. (2010). SERMs and SERMs with estrogen for postmenopausal osteoporosis.. Rev. Endocr. Metab. Disord..

[22] Kalervo Väänänen H., Härkönen P.L. (1996). Estrogen and bone metabolism.. Maturitas.

[23] Levine J.P. (2003). Long-term estrogen and hormone replacement therapy for the prevention and treatment of osteoporosis.. Curr. Womens Health Rep..

[24] Wang X., Zhen L., Zhang G., Wong M.S., Qin L., Yao X. (2011). Osteogenic effects of flavonoid aglycones from an osteoprotective fraction of Drynaria fortunei—An *in vitro* efficacy study.. Phytomedicine.

[25] Cui J., Shen Y., Li R. (2013). Estrogen synthesis and signaling pathways during aging: From periphery to brain.. Trends Mol. Med..

[26] Xu W., Ni C., Wang Y., Zheng G., Zhang J., Xu Y. (2021). Age-related trabecular bone loss is associated with a decline in serum Galectin-1 level.. BMC Musculoskelet. Disord..

[27] Hopwood B., Tsykin A., Findlay D.M., Fazzalari N.L. (2009). Gene expression profile of the bone microenvironment in human fragility fracture bone.. Bone.

[28] Shi S., Lu C., Tian H., Ren Y., Chen T. (2020). Primary Aldosteronism and Bone Metabolism: A Systematic Review and Meta-Analysis.. Front. Endocrinol. (Lausanne).

[29] Li Y., Jin D., Xie W., Wen L., Chen W., Xu J., Ding J., Ren D. (2018). PPAR-γ and Wnt Regulate the Differentiation of MSCs into Adipocytes and Osteoblasts Respectively.. Curr. Stem Cell Res. Ther..

[30] Qadir A., Liang S., Wu Z., Chen Z., Hu L., Qian A. (2020). Senile Osteoporosis: The Involvement of Differentiation and Senescence of Bone Marrow Stromal Cells.. Int. J. Mol. Sci..

[31] Giaginis C., Tsantili-Kakoulidou A., Theocharis S. (2007). Peroxisome proliferator-activated receptors (PPARs) in the control of bone metabolism.. Fundam. Clin. Pharmacol..

[32] Vundavilli H., Tripathi L.P., Datta A., Mizuguchi K. (2021). Network modeling and inference of peroxisome proliferator-activated receptor pathway in high fat diet-linked obesity.. J. Theor. Biol..

[33] Li A.C., Glass C.K. (2004). PPAR- and LXR-dependent pathways controlling lipid metabolism and the development of atherosclerosis.. J. Lipid Res..

[34] Li C.J., Cheng P., Liang M.K., Chen Y.S., Lu Q., Wang J.Y., Xia Z.Y., Zhou H.D., Cao X., Xie H., Liao E.Y., Luo X.H. (2015). MicroRNA-188 regulates age-related switch between osteoblast and adipocyte differentiation.. J. Clin. Invest..

[35] Nuttall M., Gimble J.M. (2004). Controlling the balance between osteoblastogenesis and adipogenesis and the consequent therapeutic implications.. Curr. Opin. Pharmacol..

[36] Han L., Zheng F., Zhang Y., Liu E., Li W., Xia M., Wang T., Gao X. (2015). Triglyceride accumulation inhibitory effects of new chromone glycosides from Drynaria fortunei.. Nat. Prod. Res..

[37] Toptaş B., Kurt Ö., Aydoğan H.Y., Yaylim I., Zeybek Ü., Can A., Agachan B., Uyar M., Özyavuz M.K., İsbir T. (2013). Investigation of the common paraoxonase 1 variants with paraoxonase activity on bone fragility in Turkish patients.. Mol. Biol. Rep..

[38] Yilmaz N., Simsek N., Aydin O., Yardan E., Aslan S., Eren E., Yegin A., Buyukbas S. (2013). Decreased paraoxonase 1, arylesterase enzyme activity, and enhanced oxidative stress in patients with mitral and aortic valve insufficiency.. Clin. Lab..

[39] Yılmaz N., Eren E. (2009). Homocysteine oxidative stress and relation to bone mineral density in post-menopausal osteoporosis.. Aging Clin. Exp. Res..

[40] Mazière C., Salle V., Gomila C., Mazière J.C. (2013). Oxidized low density lipoprotein enhanced RANKL expression in human osteoblast-like cells. Involvement of ERK, NFkappaB and NFAT.. Biochim. Biophys. Acta Mol. Basis Dis..

[41] Eren E., Yilmaz N., Aydin O. (2013). Functionally defective high-density lipoprotein and paraoxonase: A couple for endothelial dysfunction in atherosclerosis.. Cholesterol.

[42] Mackinnon E.S., El-Sohemy A., Rao A.V., Rao L.G. (2010). Paraoxonase 1 polymorphisms 172T→A and 584A→G modify the association between serum concentrations of the antioxidant lycopene and bone turnover markers and oxidative stress parameters in women 25-70 years of age.. Lifestyle Genomics.

[43] Hamel P., Abed E., Brissette L., Moreau R. (2008). Characterization of oxidized low-density lipoprotein-induced hormesis-like effects in osteoblastic cells.. Am. J. Physiol. Cell Physiol..

[44] Townsend S.A., Newsome P.N. (2017). Review article: New treatments in non‐alcoholic fatty liver disease.. Aliment. Pharmacol. Ther..

[45] Riker A.I., Enkemann S.A., Fodstad O., Liu S., Ren S., Morris C., Xi Y., Howell P., Metge B., Samant R.S., Shevde L.A., Li W., Eschrich S., Daud A., Ju J., Matta J. (2008). The gene expression profiles of primary and metastatic melanoma yields a transition point of tumor progression and metastasis.. BMC Med. Genomics.

[46] Hu L.P., Zhang X.X., Jiang S.H., Tao L.Y., Li Q., Zhu L.L., Yang M.W., Huo Y.M., Jiang Y.S., Tian G.A., Cao X.Y., Zhang Y.L., Yang Q., Yang X.M., Wang Y.H., Li J., Xiao G.G., Sun Y.W., Zhang Z.G. (2019). Targeting Purinergic Receptor P2Y2 Prevents the Growth of Pancreatic Ductal Adenocarcinoma by Inhibiting Cancer Cell Glycolysis.. Clin. Cancer Res..

[47] O’Sullivan S., Naot D., Callon K., Porteous F., Horne A., Wattie D., Watson M., Cornish J., Browett P., Grey A. (2007). Imatinib promotes osteoblast differentiation by inhibiting PDGFR signaling and inhibits osteoclastogenesis by both direct and stromal cell-dependent mechanisms.. J. Bone Miner. Res..

[48] Shangguan Y., Wu Z., Xie X., Zhou S., He H., Xiao H., Liu L., Zhu J., Chen H., Han H., Wang H., Chen L. (2021). Low-activity programming of the PDGFRβ/FAK pathway mediates H-type vessel dysplasia and high susceptibility to osteoporosis in female offspring rats after prenatal dexamethasone exposure.. Biochem. Pharmacol..

[49] Morán C.E., Sosa E.G., Martinez S.M., Geldern P., Messina D., Russo A., Boerr L., Bai J.C. (1997). Bone mineral density in patients with pancreatic insufficiency and steatorrhea.. Am. J. Gastroenterol..

[50] Haaber A.B., Rosenfalck A.M., Hansen B., Hilsted J., Larsen S. (2000). Bone mineral metabolism, bone mineral density, and body composition in patients with chronic pancreatitis and pancreatic exocrine insufficiency.. Int. J. Gastrointest. Cancer.

[51] Dujsíková H., Novotný I., Tomandl J., Díte P. (2010). Chronická pankreatitida a skelet.. Vnitr. Lek..

[52] Loomes K.M., Spino C., Goodrich N.P., Hangartner T.N., Marker A.E., Heubi J.E., Kamath B.M., Shneider B.L., Rosenthal P., Hertel P.M., Karpen S.J., Molleston J.P., Murray K.F., Schwarz K.B., Squires R.H., Teckman J., Turmelle Y.P., Alonso E.M., Sherker A.H., Magee J.C., Sokol R.J. (2019). Bone Density in Children With Chronic Liver Disease Correlates With Growth and Cholestasis.. Hepatology.

[53] Xu B., He Y., Lu Y., Ren W., Shen J., Wu K., Xu K., Wu J., Hu Y. (2019). Glucagon like peptide 2 has a positive impact on osteoporosis in ovariectomized rats.. Life Sci..

[54] Eriksson R., Broberg B.V. (2019). Ishøy, PL Bone Status in Obese, Non-diabetic, Antipsychotic-Treated Patients, and Effects of the Glucagon-Like Peptide-1 Receptor Agonist Exenatide on Bone Turnover Markers and Bone Mineral Density.. Front. Psychiatry.

[55] Li F., Meng F., Xiong Z., Li Y., Liu R., Liu H. (2006). Stimulative activity of Drynaria fortunei (Kunze) J. Sm. extracts and two of its flavonoids on the proliferation of osteoblastic like cells.. Pharmazie.

[56] Yang L., Zhu X.F., Wang P.P., Zhang R.H. (2013). Effects of drynariae rhizoma water-extraction on the ability of osteogenic differentiation and it’s mechanism.. Zhong Yao Cai.

[57] Yoon W.J., Islam R., Cho Y.D., Woo K.M., Baek J.H., Uchida T., Komori T., van Wijnen A., Stein J.L., Lian J.B., Stein G.S., Choi J.Y., Bae S.C., Ryoo H.M. (2013). Pin1-mediated Runx2 modification is critical for skeletal development.. J. Cell. Physiol..

[58] Bartelt A., Behler-Janbeck F., Beil F.T. (2018). Lrp1 in osteoblasts controls osteoclast activity and protects against osteoporosis by limiting PDGF-RANKL signaling., 2018, Lrp1 in osteoblasts controls osteoclast activity and protects against osteoporosis by limiting PDGF-RANKL signaling.. Bone Res..

[59] Cho E., Jin-Kyung L., Jee-Young L. (2018). BCPA {N, N′-1,4-Butanediylbis[3-(2-chlorophenyl)acrylamide]} Inhibits Osteoclast Differentiation through Increased Retention of Peptidyl-Prolyl cis-trans Isomerase Never in Mitosis A-Interacting 1.. Int. J. Mol. Sci..

[60] Bartelt A., Behler-Janbeck F., Beil F.T. (2018). Lrp1 in osteoblasts controls osteoclast activity and protects against osteoporosis by limiting PDGF-RANKL signaling.. Bone Res..

[61] Shen Z., Chen Z. (2020). Li, Z Total Flavonoids of Rhizoma Drynariae Enhances Angiogenic-Osteogenic Coupling During Distraction Osteogenesis by Promoting Type H Vessel Formation Through PDGF-BB/PDGFR-β Instead of HIF-1α/VEGF Axis.. Front. Pharmacol..

[62] Maruyama T., Jiang M., Abbott A., Yu H.M.I., Huang Q., Chrzanowska-Wodnicka M., Chen E.I., Hsu W. (2017). Rap1b Is an Effector of Axin2 Regulating Crosstalk of Signaling Pathways During Skeletal Development.. J. Bone Miner. Res..

[63] Li J., Li Y., Wang S., Che H., Wu J., Ren Y. (2019). miR-101-3p/Rap1b signal pathway plays a key role in osteoclast differentiation after treatment with bisphosphonates.. BMB Rep..

[64] Andersen T.L., del Carmen Ovejero M., Kirkegaard T., Lenhard T., Foged N.T., Delaissé J.M. (2004). A scrutiny of matrix metalloproteinases in osteoclasts: Evidence for heterogeneity and for the presence of MMPs synthesized by other cells.. Bone.

[65] Martignetti J.A., Aqeel A.A., Sewairi W.A., Boumah C.E., Kambouris M., Mayouf S.A., Sheth K.V., Eid W.A., Dowling O., Harris J., Glucksman M.J., Bahabri S., Meyer B.F., Desnick R.J. (2001). Mutation of the matrix metalloproteinase 2 gene (MMP2) causes a multicentric osteolysis and arthritis syndrome.. Nat. Genet..

[66] Jiang L., Sheng K., Wang C., Xue D., Pan Z. (2021). The Effect of MMP-2 Inhibitor 1 on Osteogenesis and Angiogenesis During Bone Regeneration.. Front. Cell Dev. Biol..

[67] Zheng X., Zhang Y., Guo S., Zhang W., Wang J., Lin Y. (2018). Dynamic expression of matrix metalloproteinases 2, 9 and 13 in ovariectomy induced osteoporosis rats.. Exp. Ther. Med..

[68] Sun B., Sun J., Han X., Liu H., Li J., Du J., Feng W., Liu B., Cui J., Guo J., Amizuka N., Li M. (2016). Immunolocalization of MMP 2, 9 and 13 in prednisolone induced osteoporosis in mice.. Histol. Histopathol..

[69] Guo L.J., Luo X.H., Wu X.P., Xie H., Zhou H.D., Zhang H., Cao X.Z., Liao E.Y. (2005). Relationships between circulating matrix metalloproteinase-1, -2 and metalloproteinase-1 levels and bone biochemical markers and bone mineral density in Chinese postmenopausal women.. Zhonghua Yi Xue Za Zhi.

[70] Yang W.S., Lee W.J., Huang K.C., Lee K.C., Chao C.L., Chen C.L., Tai T.Y., Chuang L.M. (2003). mRNA levels of the insulin-signaling molecule SORBS1 in the adipose depots of nondiabetic women.. Obes. Res..

[71] Lin W.H., Chiu K.C., Chang H.M., Lee K.C., Tai T.Y., Chuang L.M. (2001). Molecular scanning of the human sorbin and SH3-domain-containing-1 (SORBS1) gene: Positive association of the T228A polymorphism with obesity and type 2 diabetes.. Hum. Mol. Genet..

[72] Xu Y., Xin R. (2021). Sun, H Long Non-coding RNAs LOC100126784 and POM121L9P Derived From Bone Marrow Mesenchymal Stem Cells Enhance Osteogenic Differentiation *via* the miR-503-5p/SORBS1 Axis.. Front. Cell Dev. Biol..

[73] Qin S., Song G., Yu Y. (2014). Phospholipid transfer protein in diabetes, metabolic syndrome and obesity.. Cardiovasc. Hematol. Disord. Drug Targets.

[74] Scheideler M., Elabd C., Zaragosi L.E. (2008). Comparative transcriptomics of human multipotent stem cells during adipogenesis and osteoblastogenesis.. BMC Genomics.

[75] Feng H., Schorpp K., Jin J., Yozwiak C.E., Hoffstrom B.G., Decker A.M., Rajbhandari P., Stokes M.E., Bender H.G., Csuka J.M., Upadhyayula P.S., Canoll P., Uchida K., Soni R.K., Hadian K., Stockwell B.R. (2020). Transferrin Receptor Is a Specific Ferroptosis Marker.. Cell Rep..

